# Use of a Tracheal Tube as a Nasally Inserted Supraglottic Airway in a Case of Near-Fatal Airway Obstruction Caused by Epiglottitis

**DOI:** 10.1155/2019/2160924

**Published:** 2019-10-31

**Authors:** Masayuki Ozaki, Koji Murashima

**Affiliations:** Department of Anesthesia, Japan Community Health Care Organization Kyushu Hospital, Kitakyushu 806-8501, Japan

## Abstract

Airway management is critical during near-fatal obstruction of the upper airway in epiglottitis; however, this is challenging because of the sitting posture and agitated mental status of the patient. Moreover, there is currently no established protocol for safe airway management in patients with epiglottitis. Here, we describe the use of a conventional tracheal tube as a nasolaryngeal airway to maintain airway patency at the site of airway narrowing in the supine position, which enabled alleviation of imminent airway obstruction in a patient with epiglottitis. For definitive airway establishment, tracheostomy was then safely performed in the supine position.

## 1. Introduction

Acute epiglottitis is a cause of upper airway obstruction that can be fatal [1, 2]. Early interventional support of the airway is critical to avoid the onset of complete airway obstruction [3]. However, there is no established algorithm for airway management in patients with epiglottitis [4]. In such patients, there are two major obstacles to effective airway management. First, the patients are positioned in a sitting posture to maintain airway patency [5]. In the supine position, gravity may cause total airway obstruction, thereby displacing the enlarged epiglottis both posteriorly and caudally. This causes a dilemma in airway management: optimal posture for the patient causes difficulty in implementing the necessary airway procedures. Second, patients are restless and agitated because of this breathing difficulty; thus, they may be less cooperative during awake procedures [6]. It is critical to resolve these problems to achieve safe airway management in patients with near-fatal airway obstruction due to epiglottitis. Airway management was initiated in the awake state because anaesthesia induction was expected to cause complete airway obstruction. Here, we used a conventional tracheal tube as a nasally inserted supraglottic airway device to maintain airway patency in the supine position during definitive airway establishment.

## 2. Case Presentation

**Figure 1:**
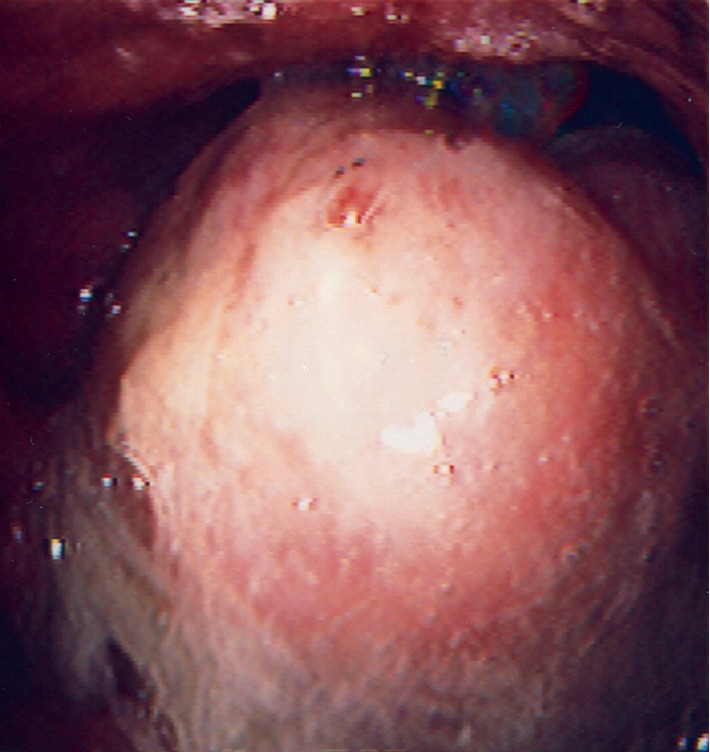
Morbidly enlarged epiglottis. The laryngeal inlet could not be observed by using the fibreoptic approach.

A 54-year-old man was admitted to the emergency department with sore throat and dysphasia. He exhibited dyspnoea and excessive salivation. Fibreoptic nasoendoscopy by the otolaryngologist revealed an inflamed and enlarged epiglottis occupying the lower pharyngeal space. Lateral soft tissue neck radiography demonstrated a prominent swollen epiglottis. The patient had to maintain a sitting position to ensure upper airway patency. At the pre-anaesthesia visit, nebulised adrenaline and intravenous steroids were applied. The use of heliox was also ordered, but it was not readily available at the emergency department. Secondary observation of the larynx revealed an enlarging epiglottis despite adrenaline and steroid administration. The patient was transferred to the operating theatre for airway management and pus drainage. Airway management was initiated in the awake state because anaesthesia induction might have led to cause complete airway obstruction. If bleeding or rupture of the epiglottitis had been observed, the alternative plan was direct laryngoscopy with rapid sequence induction; cricothyroidotomy was prepared as a backup method. Awake fibreoptic nasotracheal intubation in the sitting position was attempted with a 6.5-mm Parker Flex-Tip tube via the right nasotonsil under topical anaesthesia and sedation with fentanyl 100 *μ*g. Advancement of the fibrescope revealed a morbidly enlarged epiglottis (Figure 1), which prevented the tip of the fibrescope from proceeding into the laryngeal inlet. The anaesthetist abandoned the initial plan of awake endotracheal intubation and endeavoured to maintain upper airway patency, in order to enable use of the supine position for tracheostomy. The tracheal tube was advanced gently into the opening between the enlarged epiglottis and posterior pharyngeal wall. As the tip of the tube passed the narrowest portion of the opening, the tube bypassed the nose and paraglottic space; it then began to function as a nasolaryngeal airway. There was neither bleeding nor rupture of the epiglottic abscess, and the patient could then assume the supine position. The tracheal tube was connected to the anaesthesia circuit. Surgical tracheostomy was successfully conducted under analgesia with local lidocaine and intravenous fentanyl. Open drainage of the epiglottic abscess and left tonsillectomy were performed under general anaesthesia. The patient was subsequently admitted to the intensive care unit and fully recovered. *Prevotella melaninogenica* and *Clostridium* spp. were cultured from the pus of the abscess.

## 3. Discussion

We have described the management of a difficult airway due to epiglottitis in an agitated patient who could not assume the supine position. This nasolaryngeal airway approach prevented posterior dislocation of the epiglottis and airway obstruction in the supine position during tracheostomy. Charters and O'Sullivan defined a dedicated airway as an upper airway device dedicated to the maintenance of airway patency, prior to or during other major airway interventions [7]. Here, we have reported the supraglottic use of a nasally inserted tracheal tube as a dedicated airway for a patient with a distorted airway due to epiglottitis.

Airway patency was secured by using a nasolaryngeal airway; moreover, administration of fentanyl during the procedure ensured that the patient's mental status remained stable. It is important to control the patient's agitation during an awake procedure, as agitation can induce unanticipated movements that interfere with the surgical procedure and may cause sudden catheter displacement; displaced catheters are difficult to re-establish in the appropriate locations.

The use of heliox might have reduced the patient's respiratory workload. However, it was not readily available at the emergency department, and the airway management procedure was completed before it was delivered. Despite the use of nebulised adrenaline and intravenous steroids, the patient showed further deterioration of the epiglottitis; we presume that these treatments might be more effective during earlier phases of the disease. When the epiglottis becomes swollen, it might not be resolved in a rapid manner. Gaseous induction is another method that can be used for anaesthesia in patients with epiglottitis. However, it should be adopted in patients who can assume the supine position, because slow induction of anaesthesia does not guarantee airway patency in a patient with a morbidly swollen epiglottis.

Fortunately, no bleeding or rupture of the swollen epiglottis occurred. In case of procedural failure, the following back-up plans were in place. If the epiglottis had bled or ruptured, we would have discontinued awake intubation and performed rapid sequence induction. If laryngospasm had occurred, we would have withdrawn the fibre and connected the tube to the anaesthesia circuit, closed the other side of the nasotonsil and mouth, and supplied 100% oxygen. If hypoxia had developed with laryngospasm, we would have performed anaesthesia induction. However, we presume that potential laryngospasm might be resolved rapidly because the laryngeal closure reflex exhibits a degree of voluntary control from higher cerebral centres in the awake state [8]. If oral or nasal intubation had been failed after anaesthesia induction, we would have performed cricothyroidotomy.

In conclusion, control of the patient's posture and mental status was important during airway management in this patient, who exhibited imminent airway obstruction due to epiglottitis. The use of a tracheal tube as a nasolaryngeal airway limited the possibility of airway obstruction. The outcome of this case suggests that a conventional tracheal tube can be used as a nasally inserted supraglottic airway within acute anatomic distortions caused by epiglottitis.
